# HPV positivity despite cytological negativity: Risk assessment with colposcopic and histopathological findings

**DOI:** 10.12669/pjms.42.9.14703

**Published:** 2026-09

**Authors:** Aysegul Cakmak Madakbas

**Affiliations:** 1 Aysegul Cakmak Madakbas, MD Obstetrics and Gynecology Department, Duzce University Faculty of Medicine Hospital, Duzce, Turkey

**Keywords:** Cytology-negative, Colposcopy, Diagnostic concordance, HPV-positive, Human papillomavirus

## Abstract

**Objective:**

To evaluate agreement between colposcopy-directed histopathological findings and final histopathological diagnoses in women with negative cytology but positive high-risk human papillomavirus (HR-HPV), and to assess associations of HPV genotypes with concordance and recurrence.

**Methodology:**

This retrospective study included women aged ≥18 years with negative cytology and HR-HPV positivity who underwent colposcopy at a tertiary center between January 2020 and January 2025. Histopathological results were obtained from endocervical curettage (ECC), biopsy, or conization. Concordance was defined as agreement between colposcopy-directed histopathological findings and the final histopathological diagnosis. Patients were followed for at least 12 months, with recurrence defined as histopathological detection of LSIL/HSIL or re-detection of HR-HPV during follow-up.

**Results:**

A total of 222 women were included (median age: 44 years). Overall concordance was 53.2%. HPV16-positive women had lower concordance than HPV16-negative women (46.7% vs. 60.8%, p=0.036). HPV18, single-type, and multiple-type HR-HPV were not associated with concordance (all p>0.05). Recurrence occurred in 24.0% of patients with available follow-up data (n=125), with no significant association with HPV genotype.

**Conclusion:**

Colposcopy-directed histopathological assessment shows moderate agreement with final histopathology in cytology-negative, HR-HPV-positive women. HPV16 positivity may be associated with lower concordance, while HPV genotype was not associated with recurrence. Careful follow-up may be warranted, and larger prospective studies are needed to confirm these findings.

## INTRODUCTION

Cervical cancer remains a major global health concern and is currently the fourth most frequently diagnosed cancer among women, with more than 600,000 new cases reported in 2020.^1^ Because the disease is largely preventable, screening and early detection continue to play a crucial role. Infection with high-risk human papillomavirus (HR-HPV) is the main etiological factor, and HPV DNA can be identified in the vast majority of cervical cancer cases.^2^ Owing to the strong causal association, HPV-based screening has been adopted in many countries and is now considered comparable or, in some settings, superior to cytology.^3^

Although cytology has been used worldwide for decades, its sensitivity is limited and typically ranges between 50–60%.^4^ HPV tests, on the other hand, have much higher sensitivity (90–95%) and outperform cytology in detecting CIN2+ lesions.^5^ For this reason, women who have a negative cytology result but test positive for HPV represent a clinically important group. Even with normal cytology, a significant proportion of CIN2+ lesions may still be found.^6^

Colposcopy is the primary method for evaluating these women; however, its diagnostic performance is highly operator-dependent and may vary considerably.^7^ Misalignment between colposcopic impressions and histopathology is common, especially when lesions are subtle or poorly visualized.^8^ Understanding the virological and clinical factors that influence this concordance is therefore essential.

HPV genotypes differ considerably in their clinical behavior. HPV16 alone accounts for more than half of CIN3 lesions and the majority of cervical cancers, and is known for its aggressive nature.^9^ HPV18 has been linked more strongly to adenocarcinoma and tends to follow a different pathological pattern.^10^ Evidence on genotype-specific effects on colposcopy performance, particularly in cytology-negative patients, is limited and inconsistent across studies.^11^ HPV16-associated lesions may present with subtle or atypical colposcopic features, potentially leading to under-recognition or misclassification during visual assessment. This raises the possibility that genotype-specific biological behavior may influence the accuracy of colposcopic evaluation.

Persistent infection and recurrence also vary among HPV types. HPV16 and HPV18 are known to persist longer than other high-risk types. However, in women with normal cytology, the association between genotype and recurrence remains unclear. Multiple factors—including age, immune response, smoking, and histopathology—are believed to contribute to recurrence risk.^12^ In particular, the relationship between HPV genotype and short-term recurrence, defined as the reappearance of HPV positivity or cervical intraepithelial lesions during follow-up, remains insufficiently clarified in cytology-negative populations.

However, data evaluating both colposcopic–histopathological concordance and recurrence in cytology-negative but HPV-positive women remain limited, particularly in relation to specific HPV genotypes. Most existing studies have focused either on cytological abnormalities or on broader screening populations, leaving uncertainty regarding diagnostic performance in this clinically relevant subgroup.

Therefore, this study aimed to evaluate the agreement between colposcopy-directed histopathological findings and final histopathological diagnoses, to investigate the potential impact of HPV genotypes on this concordance, and to assess recurrence during follow-up in women with negative cytology but positive high-risk HPV.

## METHODOLOGY

This retrospective study was conducted at Duzce University Faculty of Medicine Hospital between January 2020 and January 2025. Women aged ≥18 years with high-risk HPV (HR-HPV) positivity and negative cytology who underwent colposcopic evaluation were included.

### Ethical approval:

It was obtained from the Duzce University Ethics Committee (Approval No: 2025/244, Date: 11.09.2025). Data were retrieved from the hospital electronic medical record system (MIA-MED), patient charts, and pathology reports.

### Inclusion criteria:

- Age ≥18 years, HR-HPV positivity, negative cytology obtained on the same date, documented colposcopic examination, and available histopathological results (endocervical curettage [ECC], biopsy, or conization).

### Exclusion criteria:

- HPV-negative status, cytological abnormalities of ASC-US or higher, absence of colposcopy, missing histopathological data, and a history of previously treated CIN3 or cervical cancer.

Demographic characteristics (age, parity, smoking status, menopausal status), HPV genotypes (HPV16, HPV18, single- and multiple-type HR-HPV), histopathological outcomes, and follow-up data were recorded. Because standardized visual colposcopic impression categories were not consistently available in the retrospective records, concordance was evaluated based on histopathological agreement between colposcopy-directed biopsy/ECC findings and the final histopathological diagnosis. When more than one histopathological specimen was available, the most severe diagnosis was accepted as the reference standard. Concordance was defined as agreement between the initial colposcopy-directed histopathological finding and the final histopathological diagnosis, while any upgrade or downgrade was considered discordance. Patients were followed for at least 12 months after the initial evaluation, with follow-up duration ranging from 12 to 72 months. Recurrence was defined as histopathological detection of LSIL or HSIL, or re-detection of HR-HPV positivity during follow-up.

### Statistical analysis:

Statistical analysis was performed using IBM SPSS Statistics for Windows, version 25.0 (IBM Corp., Armonk, NY, USA). Continuous variables were expressed as mean ± standard deviation or median (interquartile range), depending on distribution assessed by the Shapiro–Wilk test. Categorical variables were presented as frequencies and percentages. Associations between categorical variables were evaluated using the chi-square test or Fisher’s exact test, as appropriate. Univariate analyses were performed to assess the relationship between HPV genotypes and recurrence. Given the limited number of recurrence events (n=30), multivariable logistic regression analysis was not performed to avoid model overfitting. A p-value <0.05 was considered statistically significant.

## RESULTS

A total of 222 women with negative cytology but positive high-risk HPV were included in the study. The median age of the cohort was 44 years (IQR: 38–52), and the median parity was 2 (IQR: 1–3). Among participants, 26.6% were smokers and 21.2% were postmenopausal. HPV16 was the most common genotype, detected in 54.1% of women, followed by HPV18 in 19.8%. Single-type high-risk HPV infection was identified in 28.8% of participants, whereas multiple-type infections were present in 14.9%. Endocervical curettage (ECC) results were available for all patients, with negative or chronic cervicitis findings in 96.4%, LSIL in 0.9%, and HSIL in 2.7%. Biopsy results showed chronic cervicitis in 54.1% of women, LSIL in 26.6%, and HSIL in 19.4%. Conization was performed in 45 patients (20.3%), and HSIL was confirmed in 53.3% of these cases (Table-I).

**Table-I:** Descriptive Characteristics of the Study Population (n=222)

Characteristic	Value
Age (years)	Median 44 (IQR 38–52)
Parity	Median 2 (IQR 1–3), range 0–6
Smoking	59 (26.6%)
Postmenopausal status	47 (21.2%)
HPV16 positive	120 (54.1%)
HPV18 positive	44 (19.8%)
Single-type HR-HPV	64 (28.8%)
Multiple-type HR-HPV	33 (14.9%)
ECC result	Chronic cervicitis 214 (96.4%); LSIL 2 (0.9%); HSIL 6 (2.7%)
Biopsy result	Chronic cervicitis 120 (54.1%); LSIL 59 (26.6%); HSIL 43 (19.4%)
Conization performed	45 (20.3%)
Conization result (n=45)	Chronic cervicitis 9 (20.0%); LSIL 12 (26.7%); HSIL 24 (53.3%)
Colposcopy–histopathology concordance	118 (53.2%)
Recurrence during follow-up (n=125)	30 (24.0%)

* Values are presented as n (%) or median (interquartile range), as appropriate.

The overall concordance between colposcopic impressions and histopathological findings was 53.2%, while discordance was observed in 46.8% of cases. Discordance reflected both underestimation and overestimation by colposcopy, with underestimation appearing more frequent (Table-II).

**Table-II:** Relationship Between HPV Genotypes and Colposcopy–Histopathology Concordance (n=222).

HPV Genotype	Concordant n (%)	Discordant n (%)	p-value
HPV16 positive (n=120)	56 (46.7%)	64 (53.3%)	0.036
HPV16 negative (n=102)	62 (60.8%)	40 (39.2%)	
HPV18 positive (n=44)	21 (47.7%)	23 (52.3%)	0.421
HPV18 negative (n=178)	97 (54.5%)	81 (45.5%)	
Single-type HR-HPV (n=188)	96 (51.1%)	92 (48.9%)	0.142
Multiple-type HR-HPV (n=34)	22 (64.7%)	12 (35.3%)	

* Values are presented as n (%). Percentages are calculated within each row.

p-values were calculated using the chi-square test.

The relationship between HPV genotypes and diagnostic concordance was evaluated in all patients (n=222). HPV16-positive women demonstrated significantly lower concordance rates compared with HPV16-negative women (46.7% vs. 60.8%, p = 0.036). No statistically significant association was observed between HPV18 positivity and concordance (47.7% vs. 54.5%, p = 0.421). Similarly, single-type and multiple-type high-risk HPV infections were not significantly associated with concordance. Although multiple-type HPV infection showed a higher concordance rate compared with single-type infection (64.7% vs. 51.1%), this difference did not reach statistical significance (p = 0.142) (Table-II).

Recurrence analysis was performed in 125 patients with available follow-up data. During follow-up, recurrence was observed in 30 patients (24.0%). There was no statistically significant association between HPV genotype and recurrence. Recurrence rates were comparable between HPV16-positive and HPV16-negative groups (22.9% vs. 25.5%, p = 0.736). Similarly, HPV18 status was not associated with recurrence (23.8% vs. 24.0%, p = 0.982). Single-type and multiple-type HPV infections also showed no significant differences in recurrence rates (22.2% vs. 37.5%, p = 0.767 and p = 0.074, respectively) (Table-III).

**Table-III:** Association Between HPV Genotypes and Recurrence During Follow-up (n=125).

HPV Genotype	Recurrence n (%)	No Recurrence n (%)	p-value
HPV16 positive (n=70)	16 (22.9%)	54 (77.1%)	0.736
HPV16 negative (n=55)	14 (25.5%)	41 (74.5%)	
HPV18 positive (n=21)	5 (23.8%)	16 (76.2%)	0.982
HPV18 negative (n=104)	25 (24.0%)	79 (76.0%)	
Single-type HR-HPV (n=36)	8 (22.2%)	28 (77.8%)	0.767
Multiple-type HR-HPV (n=16)	6 (37.5%)	10 (62.5%)	0.074

* Values are presented as n (%). Percentages are calculated within each row. Analysis was performed in patients with available follow-up data. p-values were calculated using the chi-square test.

## DISCUSSION

This study evaluated the agreement between colposcopy-directed histopathological findings and final histopathological diagnoses in women with negative cytology but positive high-risk HPV, and assessed the effect of HPV genotypes on this agreement and recurrence. Our findings indicate that HPV16 positivity is associated with lower concordance, whereas HPV18 and other high-risk HPV types were not significantly associated with diagnostic agreement or recurrence.

Colposcopy remains a key tool in the evaluation of HPV-positive women, although its diagnostic performance is highly operator-dependent.^13^ Macdonald et al. reported higher colposcopic detection rates for HPV16-associated CIN2+ lesions compared to other genotypes.^14^ In addition, HPV16 is known to be associated with higher-grade lesions, persistence, and progression.^15^ Despite this, we observed lower concordance in HPV16-positive patients (46.7% vs. 60.8%, p = 0.036). This may be explained by the presence of subtle or heterogeneous lesions in HPV16 infection, which may lead to underestimation during colposcopy-directed sampling. This finding is consistent with previous studies suggesting genotype-specific differences in lesion morphology and detectability (11). HPV18 has been reported to follow a different clinical pattern compared to HPV16.^16^ In our study, HPV18 was not associated with concordance. Although HPV18 is more frequently linked to glandular lesions, which are more difficult to detect colposcopically, the limited number of such cases in our cohort may explain the lack of association.^17^ Reported concordance rates between colposcopic findings and histopathology range between 40% and 65%, and the 53.2% concordance observed in our study is consistent with the literature.^18^ Larger or more extensive lesions have been associated with HPV16 infection, which may partly explain discrepancies between initial and final histopathological findings.^19^ The management of cytology-negative but HR-HPV-positive women remains clinically important. Dai et al. demonstrated a substantial rate of CIN2+ lesions even in HPV16/18-negative patients and reported a concordance rate of 61.0%.^20^ These findings support the use of colposcopy in this population regardless of genotype. Regarding HPV multiplicity, we observed a higher concordance rate in multiple-type HPV infections compared to single-type infections; however, this difference was not statistically significant (p = 0.142). This suggests that HPV multiplicity alone may not significantly influence diagnostic agreement.

In recurrence analysis, no significant association was found between HPV genotype and recurrence. Recurrence occurred in 24.0% of patients with available follow-up data. Previous studies have reported higher recurrence and persistence rates in HPV16-positive patients after treatment.^21^ However, our cohort consisted largely of low-grade lesions and had a relatively short follow-up duration, which may explain the absence of a significant association. Although recurrence appeared numerically higher in patients with multiple-type HPV infection, this difference was not statistically significant (p = 0.074), suggesting a possible trend that requires further investigation. Discrepancies between colposcopy-directed biopsy and final histopathology may be influenced by multiple factors, including lesion characteristics, cervical anatomy, inflammation, and sampling variability.^22^

### Strengths:

The main strength of our study is the evaluation of diagnostic agreement and recurrence in a well-defined cohort of cytology-negative but HPV-positive women.

### Limitations:

First, the retrospective design may introduce selection bias. Second, standardized visual colposcopic impression categories were not consistently available, and therefore agreement was assessed based on histopathological findings rather than visual colposcopic grading. Agreement was evaluated using percent concordance, and kappa statistics were not calculated due to the lack of standardized colposcopic categories. Finally, the limited number of recurrence events and variable follow-up duration may have reduced statistical power.

## CONCLUSION

Colposcopy shows moderate agreement with histopathology in women with negative cytology but positive high-risk HPV. HPV16 positivity was associated with lower diagnostic concordance, while HPV genotype was not significantly associated with recurrence. These findings highlight the need for careful interpretation of colposcopic findings and close follow-up in this patient group. Further prospective studies are needed to confirm these results.
